# Deconvoluting low yield from weak potency in direct-to-biology workflows with machine learning†

**DOI:** 10.1039/d3md00719g

**Published:** 2024-02-15

**Authors:** William McCorkindale, Mihajlo Filep, Nir London, Alpha A. Lee, Emma King-Smith

**Affiliations:** a Cavendish Laboratory, University of Cambridge UK; b Department of Chemical and Structural Biology, The Weizmann Institute of Science Israel; c Yusuf Hamied Department of Chemistry, University of Cambridge UK esk34@cam.ac.uk

## Abstract

High throughput and rapid biological evaluation of small molecules is an essential factor in drug discovery and development. Direct-to-biology (D2B), whereby compound purification is foregone, has emerged as a viable technique in time efficient screening, specifically for PROTAC design and biological evaluation. However, one notable limitation is the prerequisite of high yielding reactions to ensure the desired compound is indeed the compound responsible for biological activity. Herein, we report a machine learning based yield-assay deconfounder capable of deconvoluting low yield from low potency to identify false negatives. We validated this approach by identifying promising SARS-CoV-2 main protease inhibitors with nanomolar activity that rivaled potency observed from the standard D2B workflow. Furthermore, we show how our framework can be utilized in a broad, *in silico* screen to produce compounds of similar potency as a D2B assay.

## Abstract 2

Augmenting direct-to-biology workflows with a new machine learning framework.

## Graphical abstract

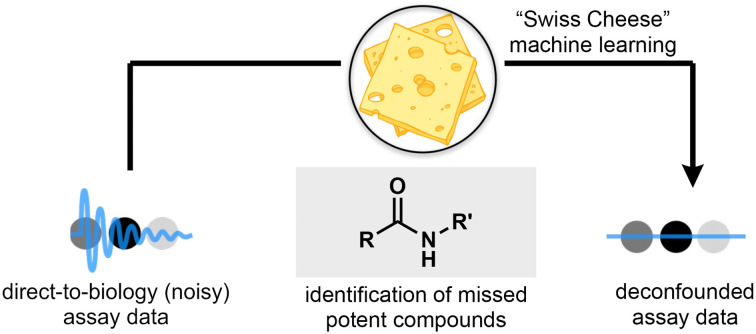

## Introduction

Direct-to-biology (D2B) in combination with high throughput screening (HTS) streamlines the target discovery pipeline by obviating the need for separation and purification. Reactive chemical fragment and PROTAC design have both significantly benefitted from this style of HTS.^1–4^ However, D2B currently requires some careful experimental design to limit the noise of crude reactions. For example, reagent scavengers may be used to remove unwanted reaction components *via* filtration, but their application is limited to select reagents. For common chemistry such as peptide bond formation reactions, resins to remove residual coupling agent are commercially available, but this is not universal for all useful reactions.^1^ Alternatively, crude reaction mixtures can be used for biological investigation, however, the reagents utilized must not interfere with the assay to form false negatives or false positives.^3^ Likewise for any byproducts formed, reaction optimization should be performed to limit the number of side products.^2^ Often only higher yielding reactions are used in assays; lower yielding reactions are not further investigated due to the confounding effects of lower yielding reactions in the biological assay. A robust method to deconvolute the inherent noise of the crude reaction from the biological activity would increase the scope of high throughput D2B to a more diverse range of chemistries.

**Fig. 1:**
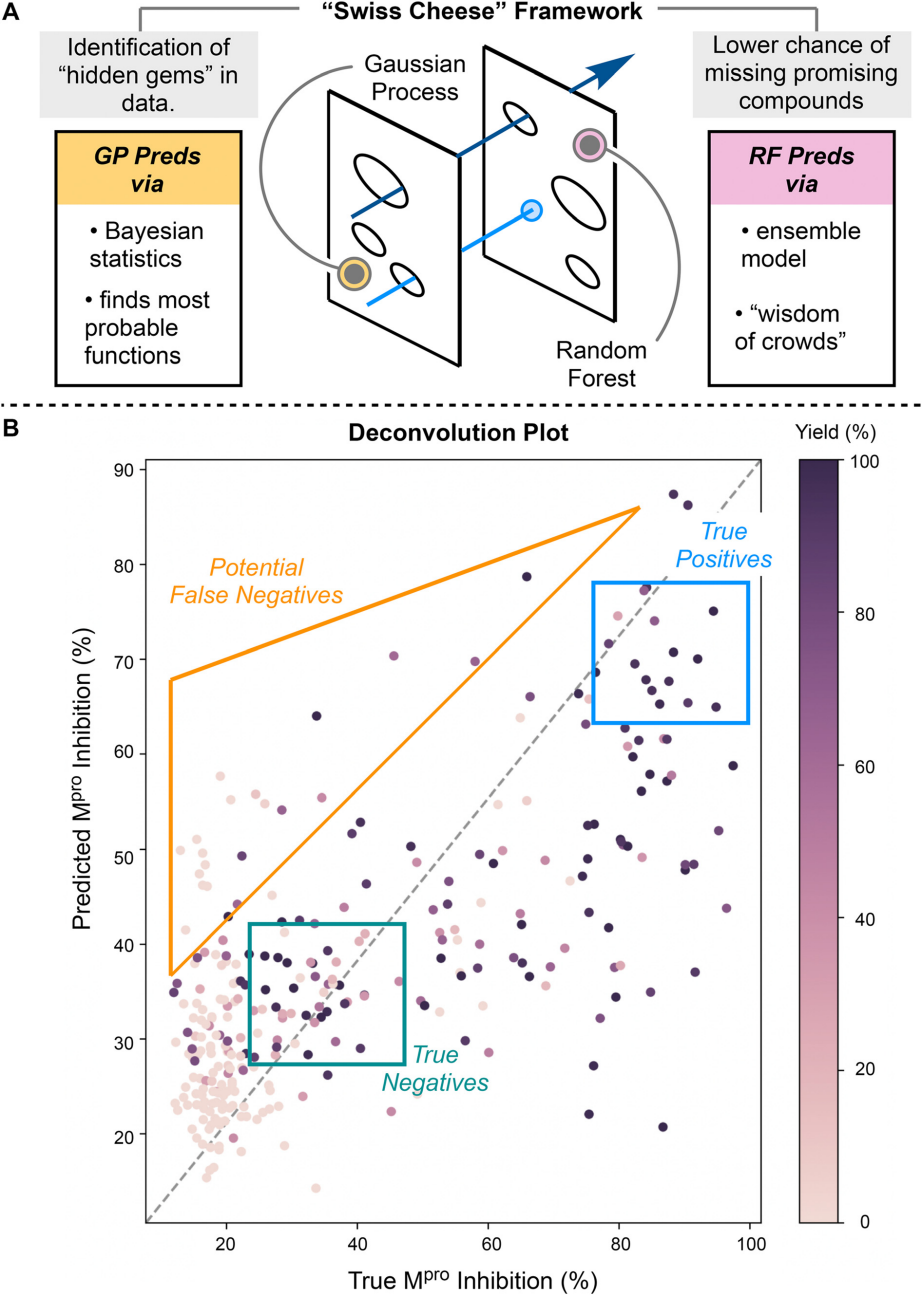
Overview of ML workflow. (A) Graphical representation of the “Swiss cheese”. Hits missed by one model could be caught by the other. (B) Analysis of a deconvolution plot. Points inside the blue box are true positives (D2B hits). Points inside the teal box are true negatives. Points inside the orange triangle are potential false negatives.

Machine learning (ML) methods have been successfully applied to predicting a variety of medicinal chemistry relevant properties including QSAR, IC_50_ prediction of purified compounds, and improved docking studies.^5–7^ The principal driving force behind these computational algorithms is identifying and subsequently modelling correlations between chemical or biological features and measured outcome. Our hypothesis is that a machine learning approach can effectively act as a denoising algorithm – picking out average trends in structure–activity relationships across chemical space amid noise such as variations in yield. Prior reports utilizing ML to identify data outliers, including false negatives, have encompassed a variety of approaches in the chemical and biological areas. Clustering algorithms such as principal component analysis (PCA), *k*-nearest neighbors (KNN), or hierarchical clustering are often employed.^8–11^ These methods allow for visual identification of outliers, distinguished by their literal graphical position of lying outside of the algorithmically-formed, dense cluster centers. Analytical chemistry and metabolomics have also employed the use of robust regression and classification models such as support vector machines (SVMs) and Adaboost to not only accurately model noisy systems but also to identify these deviating datapoints.^12,13^ However, these detection methods typically have two defined end points. The first is to validate the robustness of their data as low outlier levels is indicative of high-quality data. The second is to treat outliers as unwanted noise and proceed with outlier removal for clearer modelling and prospective assessment. We believe that outliers, specifically false negatives, can be harnessed for further exploration when used in combination with high throughput D2B. The false negatives of a screen are molecules which, due to experimental noise, were deemed poorly performing in their associated assay, but in reality, are actually promising lead compounds. Herein, we report a “Swiss cheese” ML approach that utilizes two distinct ML paradigms to model confounded crude reaction mixture assay data (Fig. 1). Their combined predictions allowed for identification of false negative molecules that would have been missed in a traditional D2B workflow. Additionally, this paradigm can be put towards high throughput *in silico* screening of vast molecular space to identify new molecular scaffolds.

## Results & discussion

For this investigation, we targeted the SARS-CoV-2 main protease, M^pro^, a non-structural protein that plays a key role in viral replication. M^pro^ has been noted by several groups to be a promising target for drug design due to its importance in SARS-CoV-2's lifecycle and lack of related homologues in humans which has resulted in the success of approved SARS-CoV-2 antiviral treatments, nirmatrevir (Paxlovid) and ensitrevir (Xocova).^14,15^ Numerous high throughput assays to quantitatively measure M^pro^ inhibition for rapid fragment screening have been developed.^16^ We opted for a previously reported fluorogenic assay which utilizes the synthetic fluorogenic M^pro^ substrate, [5-FAM]-AVLQSGFR-[Lys(Dabcyl)]-K-amide.^17^

Prior effort from the COVID Moonshot consortium resulted in the rapid discovery of an orally bioavailable, nanomolar inhibitor of M^pro^, 1, through the use of crowdsourcing, ML-directed synthetic complexity scores, and computationally guided structure activity relationship (SAR) screening. The authors noted the importance of the isoquinoline motif for M^pro^ P1 pocket activity, but a high tolerance of structural variation of the M^pro^ P2 pocket binding motifs. This allowed for the simplification of the original chromane ring to 1's chloro-tetrahydroisoquinoline, which furnished an N-centered handle for further SAR. Library-based derivatization of this handle with Schotten–Baumann sulfomidation resulted in a promising M^pro^ inhibitor drug candidate with improved enzymatic inhibition, antiviral activity, and improved oral bioavailability, highlighting the importance of this tail.^17^ We began a further investigation upon the N-centered handle through the formation of similarly stable amide bonds with the fast turnaround of D2B screening, but including low yielding reactions that would be traditionally discarded. We hypothesized that ML-guided hit identification would be able to identify promising scaffolds even in the noisy environment of low yielding, crude reaction mixtures. Low yielding but potent compounds will still register as low activity molecules. To this end, we synthesized a library of derivatives *via* EDC mediated peptide bond couplings (Fig. 1B). Given high throughput D2B is known for peptide bond formations, we believed this chemistry to be a good test-case for our deconfounding strategy. Thus, 300 amines were chosen, coupled to the commercially available M^pro^ P1, P2 binder, acid 2, and subjected without further purification or separation to fluorogenic M^pro^ assay.

To identify these false negatives, we designed a “Swiss cheese” ML-based deconfounder. This was achieved by selecting two distinct ML techniques known to be highly accurate in molecular property prediction but orthogonal in their methods of generating predictions. The rationale was that ML techniques that model with different algorithms would result in a robust framework more accurate than if just one or the other was used. Hits missed by one algorithm still had the potential to be identified by the other. Agreement of predictions from both models indicated a high likelihood of experimental reality. A Random Forest regressor and Gaussian Process regressor were chosen as the two ML “Swiss cheese” slices (Fig. 1A). A Random Forest is an ensemble model technique formulated from a collection of decision trees.^18^ They encapsulate the “wisdom of crowds” where a single individual decision tree may be inaccurate, but their aggregate predictions are quite accurate. This mimics the well-known phenomenon that individuals can be inaccurate at guessing the correct answer, but the collective group has remarkable accuracy. Accurate prediction of compound solubility, quantitative structure–activity relationships (QSAR), and toxicity have been achieved with Random Forest modelling.^19–21^ Contrastingly, Gaussian Processeses are based upon Bayesian statistics which predicts a distribution of functions whose sole restriction is that they pass through the known (experimental) datapoints. The properties of these functions are determined *via* the kernel, which characterizes the smoothness, periodicity, and shape of the underlying family of functions. Gaussian Processes have the additional benefit of predicting value and uncertainty. Intuitively, high uncertainty of a predicted value occurs when, at that given input, the range of values from the possible functions (determined from the kernel and known data points) is high. Gaussian Processes have accurately predicted lipophilicity, ADMET properties, and PROTAC potency in prior reports.^6,22,23^ Both models are non-parametric, making them ideal ML models for our “Swiss cheese” framework.

Whilst ML-based regression models that predict IC_50_ values from pure substrate have been validated numerous times previously, we believe these methods are less suitable in the context of D2B.^20,24–26^ To develop any model that predicts the IC_50_ outcomes (values from regression, or active/inactive from classification) of pure substrate, pure substrate must first be obtained.^27^ However, two key features of D2B are 1) the utilization of crude, unchromatographed compound in biological assays and 2) the ability to perform ultra-high throughput screens on nanogram quantities of substrate.^4^ Both of these key features make the traditional ML IC_50_ prediction platform incompatible. It would necessitate several hundred compounds be made on larger scales to ensure enough material for isolation and subsequent IC_50_ evaluation; a slower and less high throughput method which runs contrary to the goals of D2B. Thus, we propose that our model, with its capacity for false negative detection as a consequence of training upon crude IC_50_ data, lends itself as a better fit into D2B campaigns.

### Deconvolution for false negative identification

**Fig. 2:**
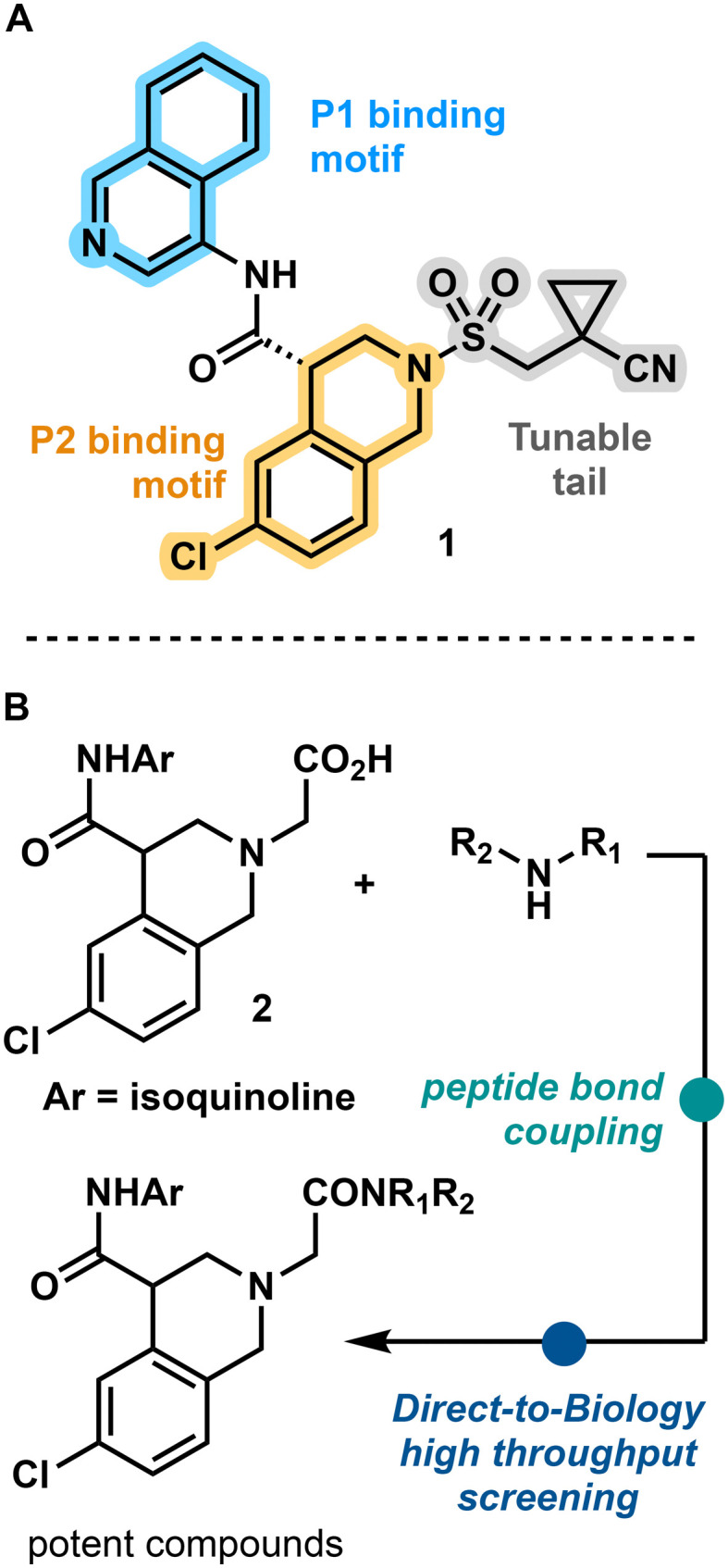
Introduction to a new SARS-CoV-2 main protease (M^pro^) inhibitor. (A) Structure of promising crowdsourced M^pro^ inhibitor. (B) Workflow for investigation of tunable tail fragment.

**Fig. 3:**
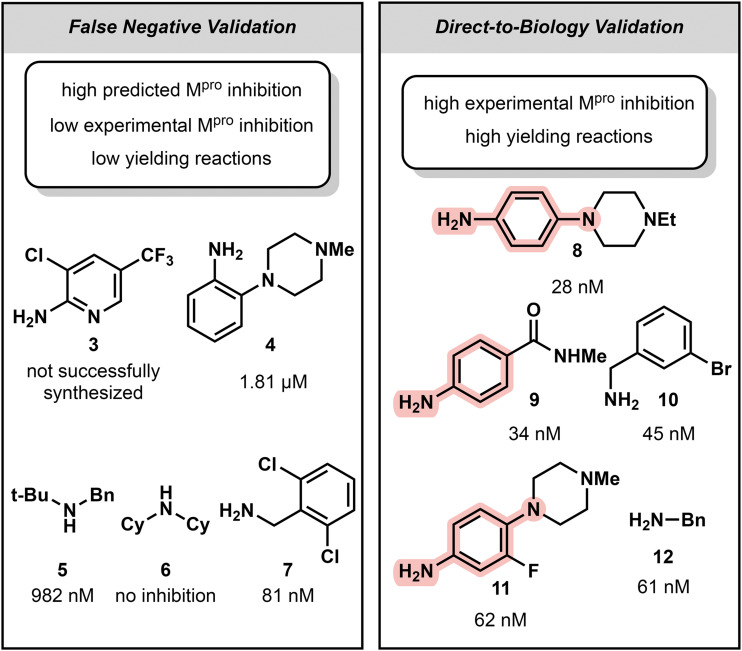
Top validated amides (the amine used in the peptide bond forming reaction shown for clarity) of crude assay inhibition. IC_50_ values of the resulting amide shown below each compound. Selection criteria for compounds is listed. (Left panel) Top 5 putative and uncovered false negatives. (Right panel) Top 5 D2B hits from crude fluorogenic assay results (true positives). Deleterious aniline motif is highlighted in pink.

We first began by identifying false negatives, these so-called “hidden gems”. These are compounds which, by nature of their low yield, have been labeled with low inhibitory activity. In a traditional D2B workflow, these compounds would be either a) excluded prior to the assay screening due to their low yields or b) require some level of scavenger-assisted separation to normalize the concentration of the potentially active compounds. Gaussian Process and Random Forest regressors were individually trained on the experimental M^pro^ inhibition data from the 300 crude amides (Fig. 2B, Table S1†). Due to the conserved nature of the acid, the Morgan fingerprint of the amine moiety was used as the featurization of each compound. The predictions for both models, visually, lead to an easy identification of true positives and true negatives: molecules that have high yields and high or low activity, respectively. However, we can also identify potential false negatives (“hidden gems”): compounds that were determined by to be structurally represented in the high activity regime, but experimentally determined to have low yield and low inhibitory activity (Fig. 1B, S1 and S2†). The top 5 compounds that were determined to meet the aforementioned criteria (high predicted activity by both Gaussian Process and Random Forest, low experimental activity, low yield as determined by LC/MS) were selected as promising false negatives. These compounds, 3–7, had reported less than 35% M^pro^ inhibitory activity *via* the fluorogenic assay and a yield under 50%. Of the five compounds, 4 were able to be synthesized in suitable purity. Whilst compound 6 was indeed inactivate (deconvolution failed), compounds 4 and 5 showed high nanomolar to low micromolar inhibitory activity and 7 and boasted an IC_50_ value of 81 nM, ranking it within the top 20 most potent D2B hits (Fig. 3 and S3†). This result showcased the ability of the “Swiss cheese” framework to find new promising molecules that would be typically bypassed. Considering the ease of implementation and the low data requirement to run the models, we believe it is a low risk, high reward strategy to augment established D2B pipelines.

### High throughput computational deconvolutional screening

Finally, we sought to investigate the potential of our “Swiss cheese” framework towards *in silico* screening. A set of 61 814 amines commercially available from Enamine were initially sourced and screened for structural alerts. Molecules with more than a single stereocenter were removed to avoid downstream complications in assessing potency. This resulted in 58 082 1° and 2° amines which were subjected to virtual screening by our trained “Swiss cheese” framework to identify possible amides with potent M^pro^ inhibition, irrespective of their potential coupling yield. The top 20 compounds predicted by the “Swiss cheese” to have high M^pro^ inhibition were synthesized and assessed (Fig. S4 and S5†). Of these compounds, 19 were successfully formed in suitable purity, and 7 of them had nanomolar inhibitory activity that ranked them in the 96th percentile of hits from the initial 300 amine screen. This resulted in an increase of sub 100 nanomolar inhibitors from 12 to 19, a 58% increase with minimal screening and computational cost.

**Fig. 4:**
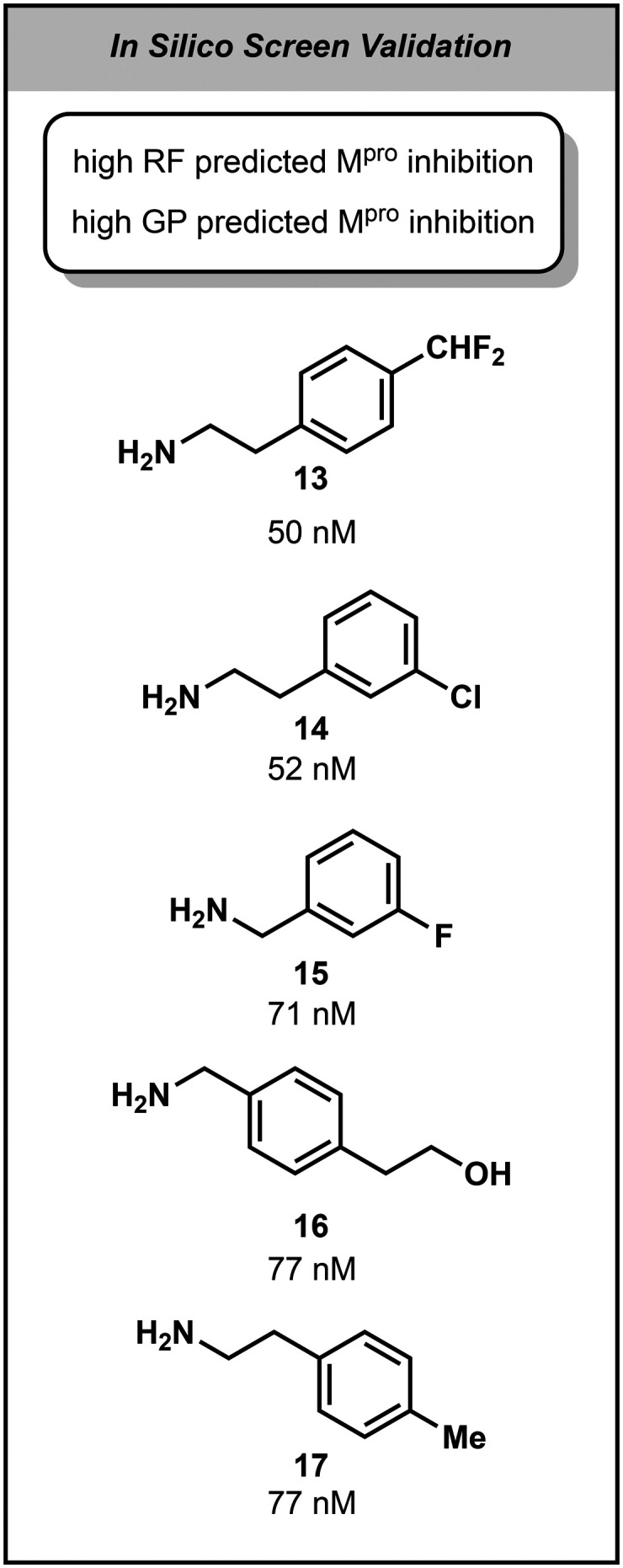
The top 5 validated amides (the amine used in the peptide bond forming reaction is shown for clarity) from the “Swiss cheese” model of the *in silico* screen of 58 000+ Enamine amines. IC_50_ values of the resulting amide shown below each compound.

Notably the structure of the top 2 most potent D2B compounds, 8 and 9, are remarkably different from the *in silico* screen's top two compounds, 13 and 14. Whilst 8 and 9 both contain aniline motifs, typically avoided due to their propensity to form reactive metabolites,^28^ this deleterious functionality is absent in not just 13 and 14, but all the top 5 predicted most potent compounds. Comparing the top *non-aniline* containing D2B hits revealed that this brief *in silico* screen resulted in compounds with similar nanomolar M^pro^ inhibition, at 50 and 52 nM respectively, compared to 45 nM and 61 nM, highlighting this framework's capabilities not only in uncovering potent compounds that would have been discarded in a traditional D2B pipeline, but also as a valuable high throughput *in silico* screening module (Fig. 4).

## Conclusion

We have showcased a dual machine learning framework that can expand the scope of D2B-compatiable reactions by deconvoluting reaction yield and potency, allowing for low yielding reactions to be included. The “Swiss cheese” framework acted as a deconfounder of low yield from low M^pro^ inhibition and resulted in the elucidation of three active compounds previously labeled as inactive. Additionally, we highlighted the framework's utility in a much broader screen of over 58 000 compounds; it would be challenging to validate such a number of compounds in a time- and cost-efficient manner. This *in silico* screen identified 7 new nanomolar potent compounds with valuable and non-deleterious structural motifs. We hope that future D2B and/or *in silico* screens can take advantage of this lightweight ML architecture which can be used in both low and high data environments to uncover new biologically valuable compounds and motifs.

## Data availability

Code for this paper and the associated data can be found at: https://github.com/wjm41/deconvoluting_low_yield.

## Author contributions

WM and EKS performed the machine modelling. MF performed the biological assays. WM, NL, AAL, and EKS conceived of the work. EKS wrote the manuscript and all authors contributed to its editing.

## Conflicts of interest

AAL is a co-founder and owns equity in PostEra Inc and Byterat Ltd. NL currently serves on the scientific advisory board of Monte Rosa Therapeutics, Larkspur Biosciences and Tesseract Medicines. WL, MF, and EKS declare no conflicts of interest.

## Supplementary Material

MD-015-D3MD00719G-s001
