# Supplementary material for: Deconvoluting low yield from weak potency in direct-to-biology workflows with machine learning
Source: RSC Med Chem. 2024 Feb 15;15(3):1015–21. doi: 10.1039/d3md00719g (PMC10953487; doi:10.1039/d3md00719g)
Supplement: MD-015-D3MD00719G-s001 [file MD-015-D3MD00719G-s001.pdf]

Electronic Supplementary Material (ESI) for RSC Medicinal Chemistry.
This journal is © The Royal Society of Chemistry 2024

Supporting Information

Deconvoluting Low Yield from Weak Potency in Direct-to-Biology Workflows with Machine Learning

William McCorkindale, Mihajlo Filep, Nir London, Alpha A. Lee, and Emma King-Smith*

Correspondence to: esk34@cam.ac.uk

Table of Contents

General Materials and Methods.. page S3
Code and Data Availability.. page S3
Peptide Bond Coupling General Procedure.. page S3
General Fluorogenic Assay Procedure... page S3-S4
Modelling, Training, and Validation Methodology... page S4
Table S1: Model metrics for each model... page S5
Figure S1: Gaussian Process Model Predictions... page S6
Figure S2: Random Forest Model Predictions.. page S7
Figure S3: Structures of Direct-to-Biology Hits.. page S8
Figure S4: Structures of top *in silico* compounds.. page S9

Figure S5: Distribution of predicted IC_{50} values.. page S10
Dose response curves for isolated compounds.. page S11-S14
Supplemental References.. page S15

General Materials and Methods:

Unless otherwise noted, all chemicals and reagents for chemical reactions were purchased at the highest commercial quality and used without further purification. Random Forest and Gaussian Process models used were run with default scikit-learn parameters (version 1.0.2). Morgan fingerprints of the amine fragments were created using the default parameters on RDKit (version 2020.09.1).

Code and Data Availability:[1]

The associated code can be found at: https://github.com/wjm41/deconvoluting_low_yield.

Peptide Bond Coupling General Procedure:

The amide library was made by reacting the carboxylic acid under the optimized reaction conditions (2 eq. amine; 2 eq. EDC; 2 eq. HOAt; 5 eq. DIPEA; DMSO; RT; 24h) with 300 amines (202 aromatics, 49 primary, and 49 secondary aliphatic amines). For library production, we used Echo LDV plates and an Echo 555 acoustic dispenser for liquid handling. Plate copies were made after diluting the reaction mixture with 4 μL DMSO. For yield estimation, 1 μL of the diluted library was transferred to an LC/MS-ready 384-well plate, followed by dilution with 20% acetonitrile in water to the final volume of 50 μL. The desired product was identified in 60% of wells.

General Fluorogenic Assay Procedure:[2]

Compounds were seeded into assay-ready plates (Greiner 384 low volume, cat. no. 784900) usingan Echo 555 acoustic dispenser, and dimethylsulfoxide (DMSO) was back-filled for a uniformconcentration in assay plates (DMSO concen-tration maximum 1%) Screening assays wereperformed in duplicate at 20mM and 50mM. Hits of greater than 50% inhibition at 50 mM were confirmed by dose response assays. Dose response assays were performed in 12-pointdilutions of twofold, typically beginning at 100 mM. Highly active compounds were repeated in a similar fashion at lower concentrations beginning at 10mM or 1 mM.

Reagents for Mpro assay were dispensed into the assay plate in 10 ml volumes for a final volume of 20 mL. Final reaction concentrations were 20 mM HEPES pH = 7.3, 1.0 mM TCEP, 50 mM NaCl, 0.01% Tween-20, 10% glycerol, 5 nM M^{pro}, and 375 nM fluorogenic peptide substrate ([5-FAM]-AVLQSGFR-[Lys(Dabcyl)]-K-amide). M^{pro} was preincubated for 15 min at room temperaturewith compound before addition of substrate and a further 30 min incubation. Protease reaction was measured in a BMG Pherastar FS with a 480/520 excitation/emission filter set. Raw data were mapped and normalized to high (Protease with DMSO) and low (No Protease)controls using Genedata Screener software. Normalized data were then uploaded to CDD Vault (Collaborative Drug Discovery). Dose response curves were generated for IC_{50} using nonlinear regression with the Levenberg–Marquardt algorithm with minimum inhibition = 0% and maximum inhibition = 100%. The assay was calibrated at different enzyme concentrations to confirm linearity and response of protease activity, as well as optimization of buffer components for most stable and reproducible assay conditions. Substrate concentration was chosen after

titration to minimize saturation of signal in the plate reader while obtaining a satisfactory and robust dynamic range of typically five- to six-fold overcontrol without enzyme. As positive control, under our assay condition, nirmatrelvir has IC_{50} of 2.6 nM.

Modelling, Training, and Validation Methodology:

The Gaussian Process (GP) and Random Forest (RF) models were trained using a dataset comprising 300 SMILES-inhibition readings from a high-throughput, direct-to-biology assay. The objective was to model inhibition as a regression problem, aiming to minimize the root-mean-square error between the models' predictions and the experimental data by employing an L2 loss function. Given the limited size of the dataset, we adopted a leave-one-out cross-validation approach to achieve a reliable estimation of the models' generalization error. In this method, the machine learning model is trained on all but one data point (i.e., 299 in our case) and then makes a prediction for the excluded data point. This process is iterated for each data point in the dataset, with the results presented in Figure 1.

Both models leverage Morgan fingerprint features with a radius of 2 and 2048 bits for molecular representation. To identify the optimal hyperparameters for the models, such as the GP kernel bandwidth and the number of RF estimators, we utilized 5-fold cross-validation implemented through the GridSearchCV function in scikit-learn.

Model	MAE	RMSE	Spearman Correlation
Random Forest	12.9	18.6	0.62
Gaussian Process	15.8	21.6	0.50
"Swiss Cheese" Model	13.4	18.7	0.60

Table S1: Model metrics for each model. Our "Swiss Cheese" model is the mean of the Random Forest and Gaussian Process models.

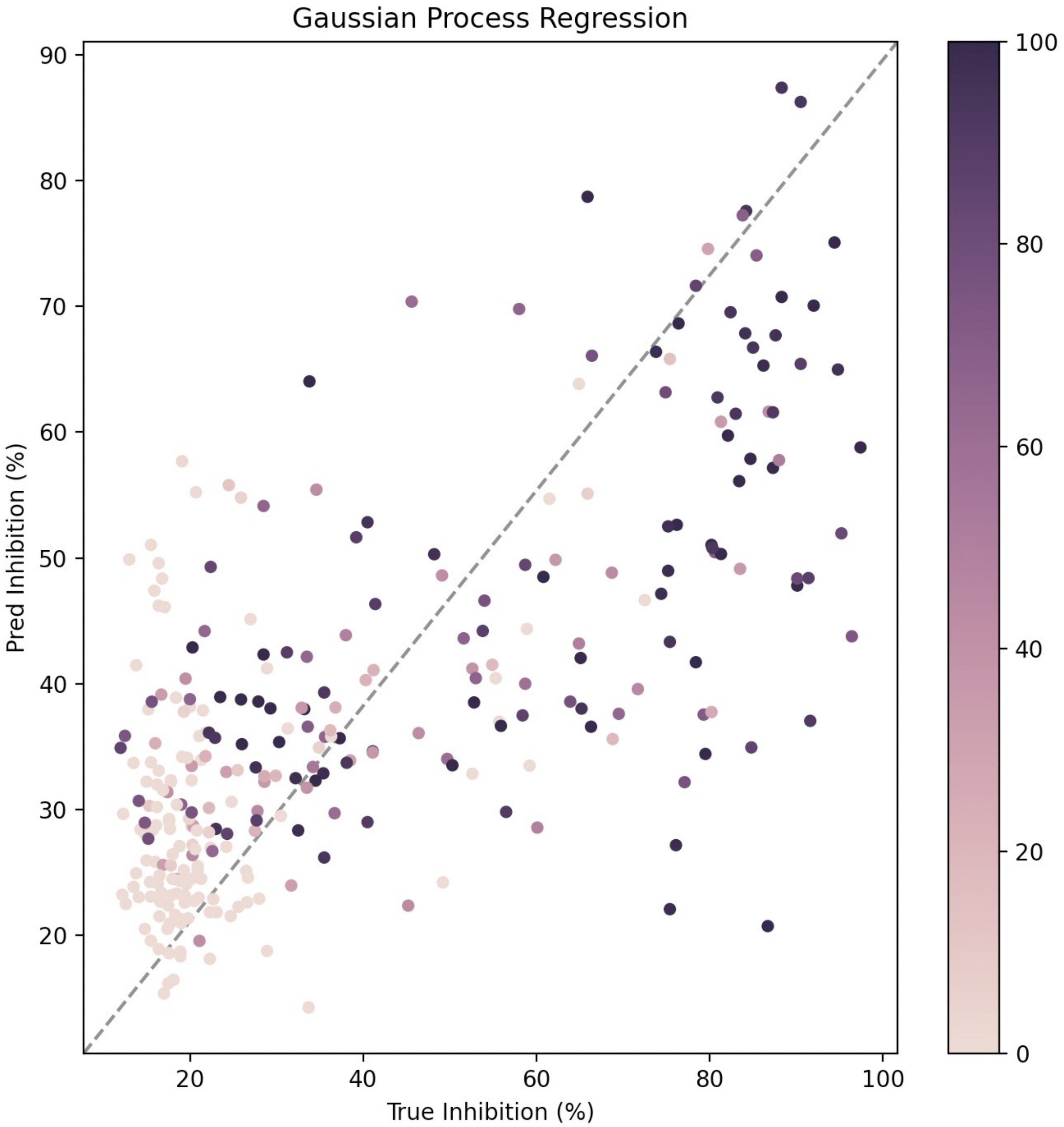

Figure S1: Gaussian Process regression results of initial 300 amide modelling. Each dot represents one potential M^{pro} inhibitor. Dotted diagonal line represents perfect model accuracy. Dot color corresponds to yield.

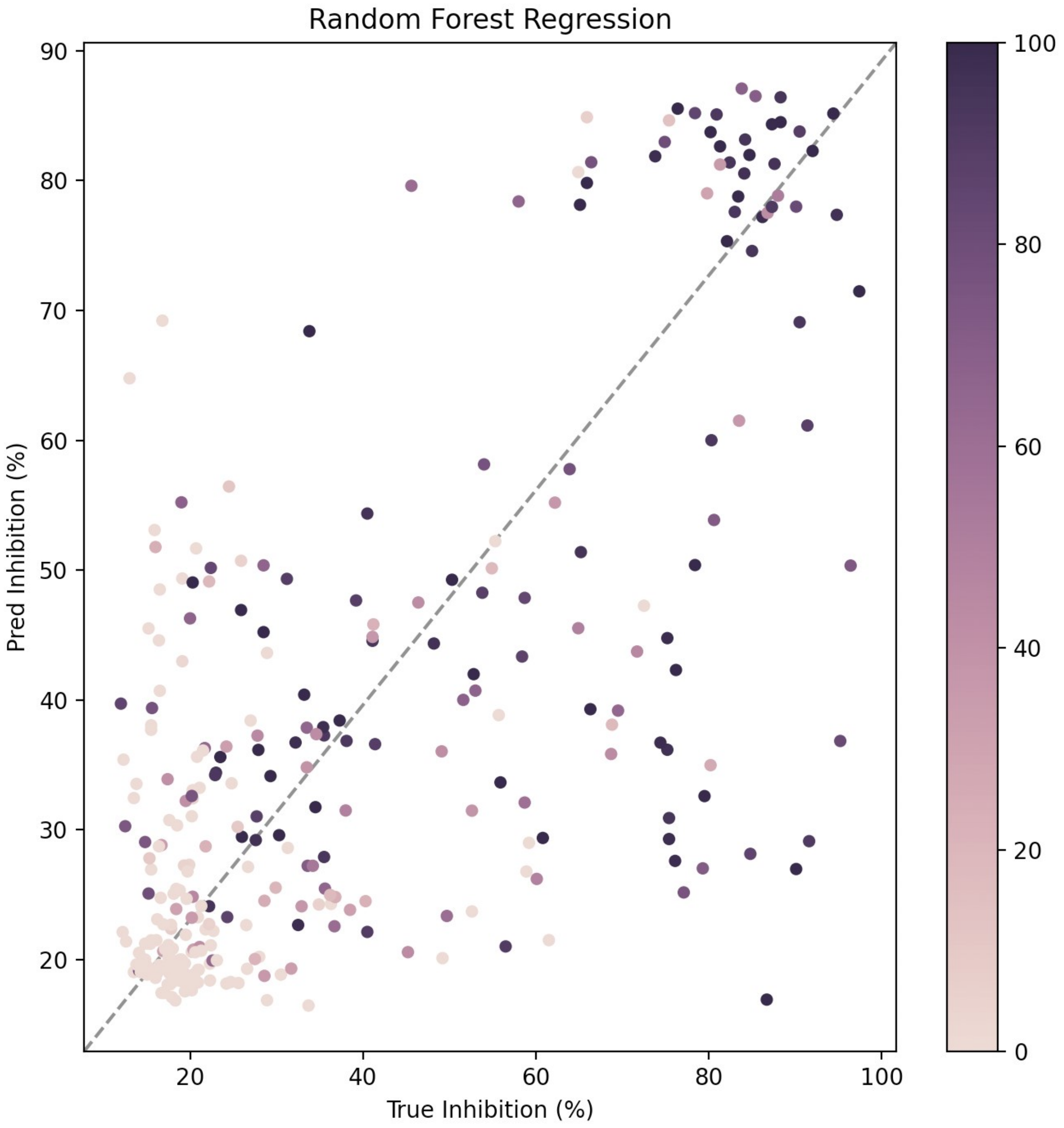

Figure S2: Random Forest regression results of initial 300 amide modelling. Each dot represents one potential M^{pro} inhibitor. Dotted diagonal line represents perfect model accuracy. Dot color corresponds to yield.

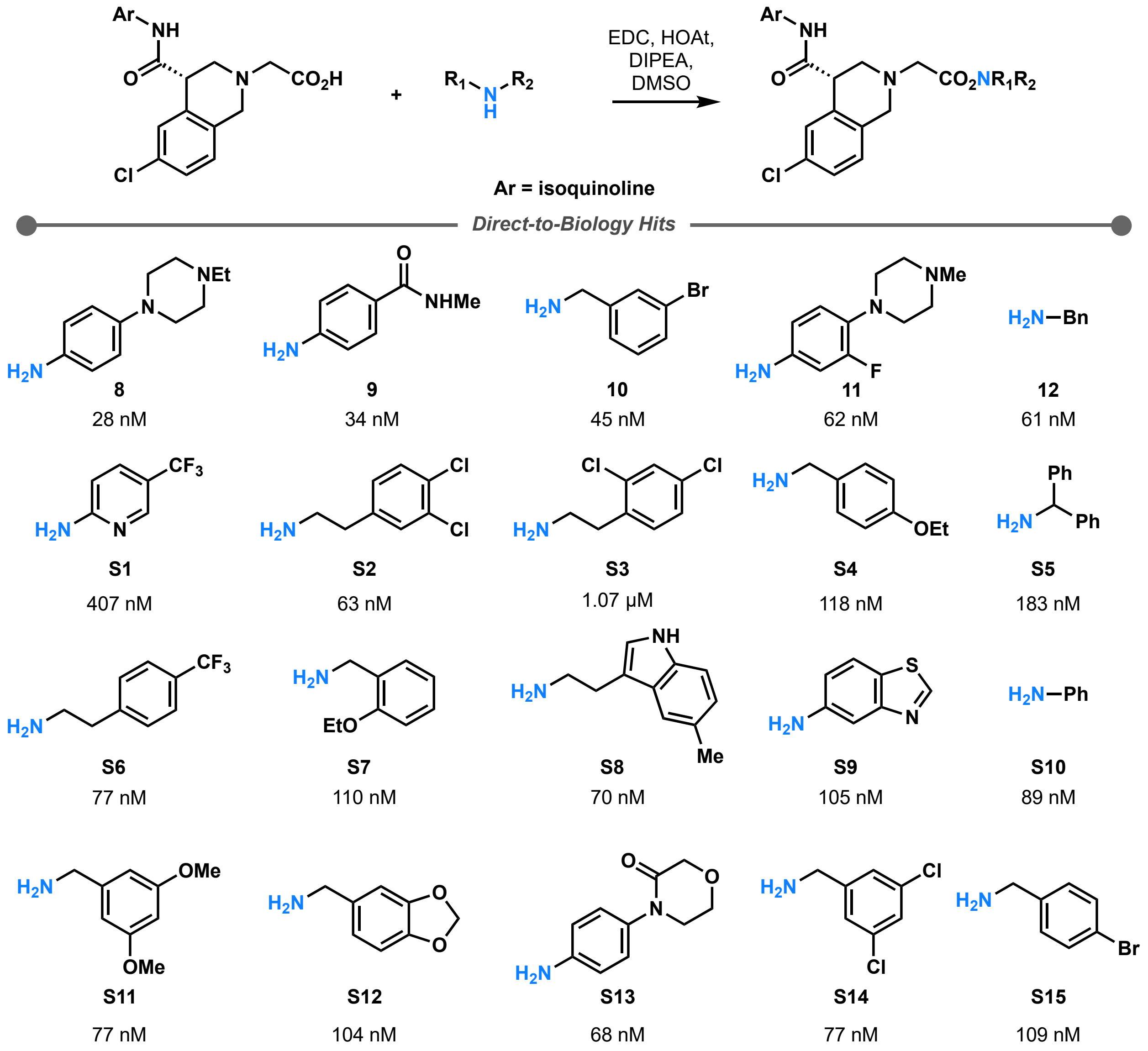

Figure S3: IC_{50} values for the top 20 direct-to-biology amides hits formed through the shown amines. Coupling location is highlighted in blue.

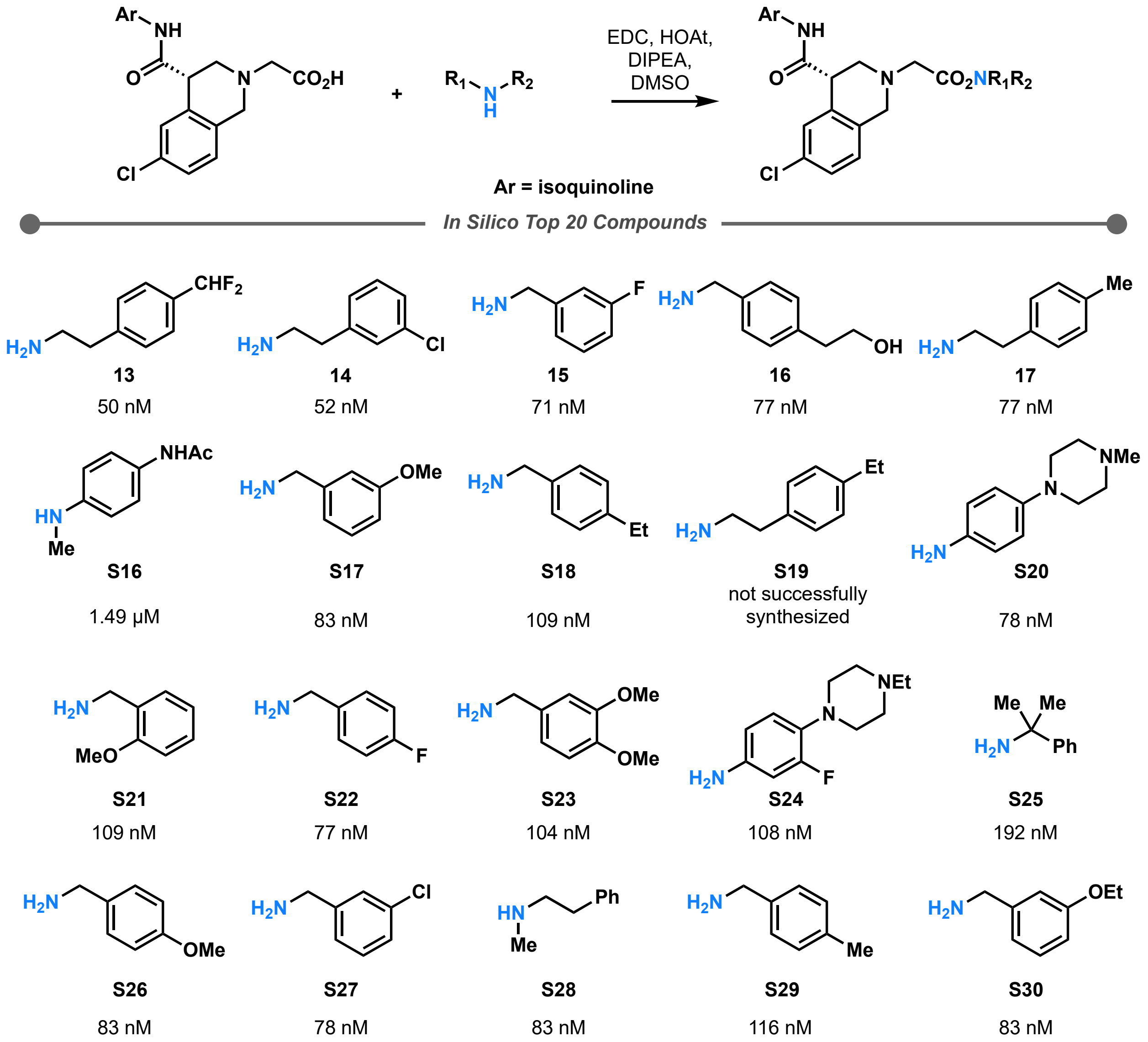

Figure S4: IC_{50} values for the top 20 most potent compounds as determined by the "Swiss Cheese" model of the *in silico* screen. Coupling location is highlighted in blue.

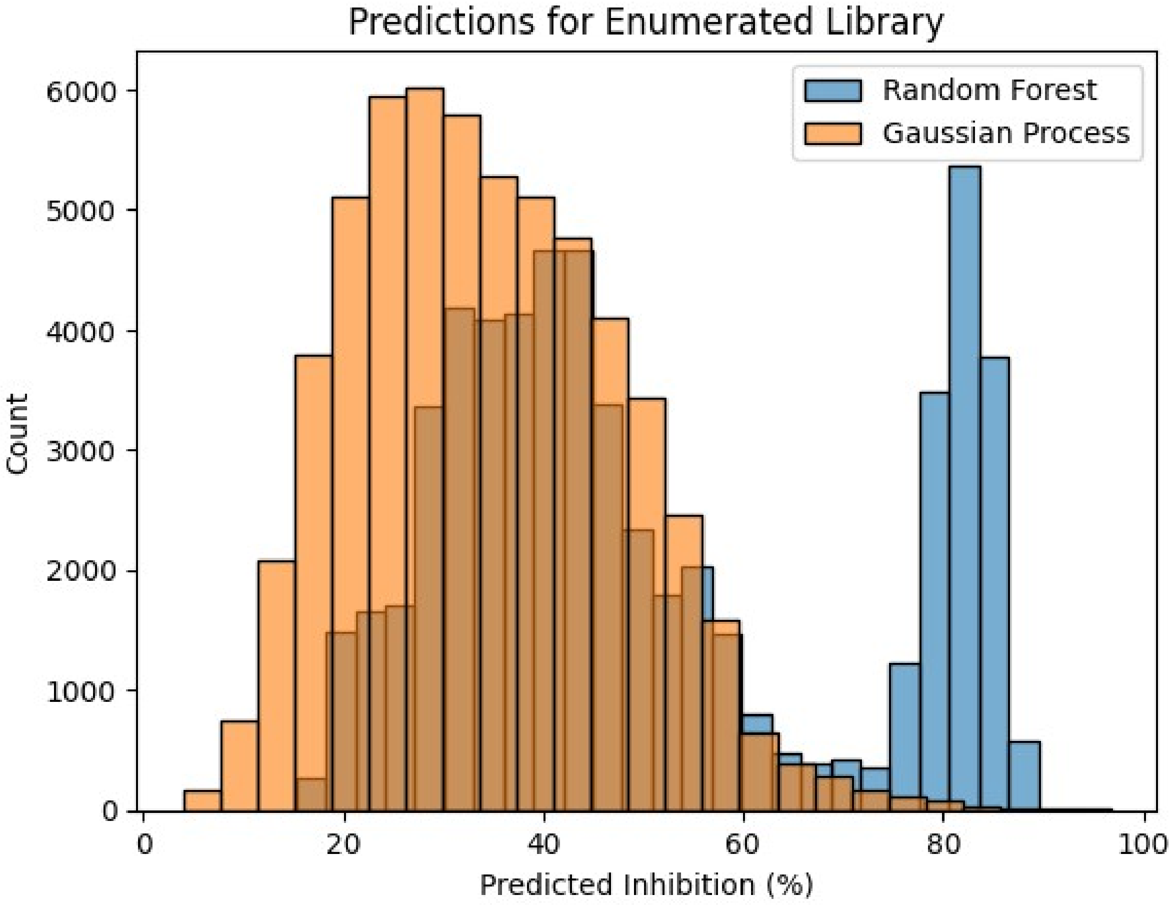

Predicted Inhibition Threshold	Number of Compounds*	Percentage of Dataset
50%	16,576	28.5%
70%	1,676	2.9%
80%	140	0.24%

* = Number of compounds from combined “Swiss Cheese” model (mean of GP & RF).

Figure S5: Distribution of predicted IC_{50} values from the Random Forest (RF) and Gaussian Process (GP) models. The number of compounds predicted by our "Swiss Cheese" model to have inhibition above 50%, 70%, and 80% are listed below.

Dose Response Curves for Isolated Compounds:
Associated curve is on the left.

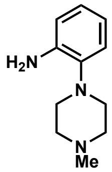

4

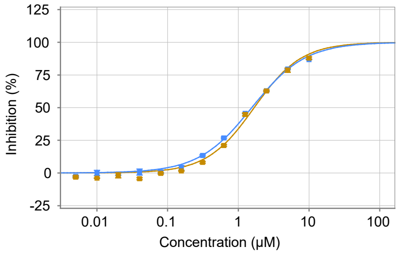

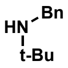

5

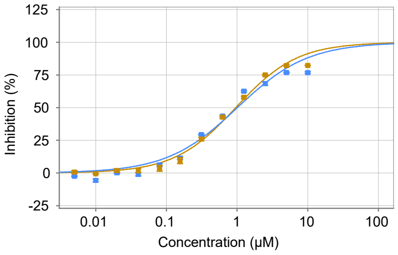

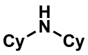

6

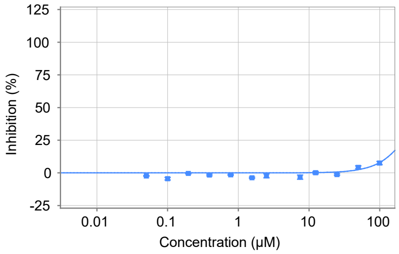

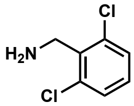

7

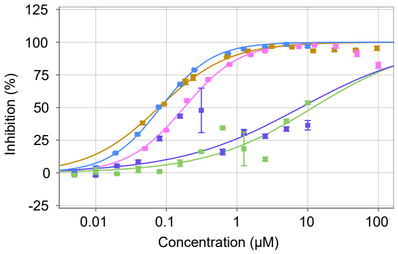

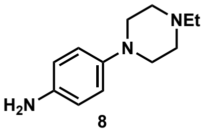

8

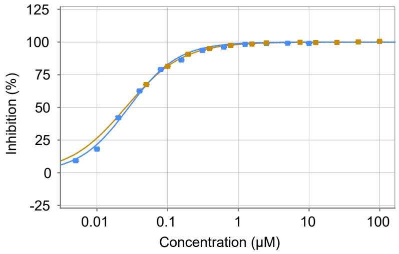

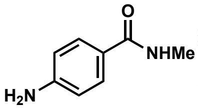

9

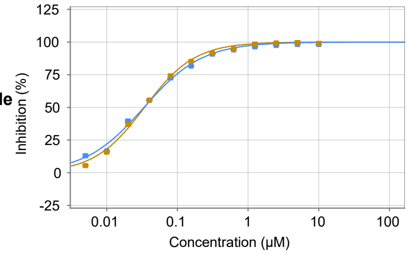

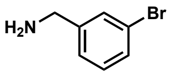

10

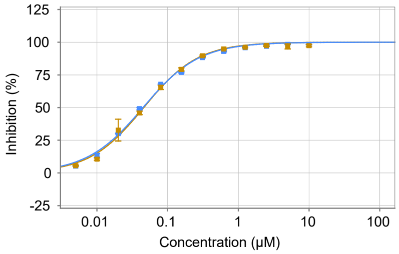

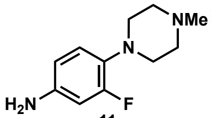

11

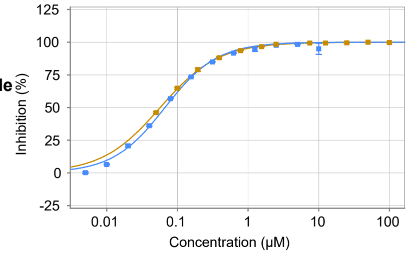

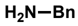

12

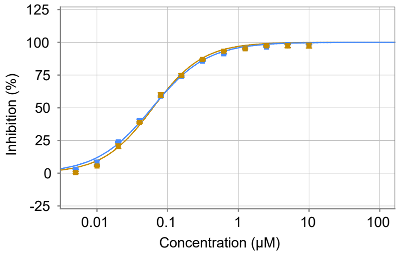

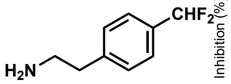

13

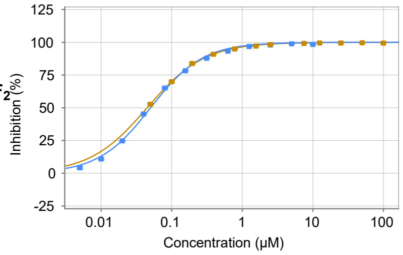

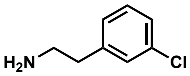

14

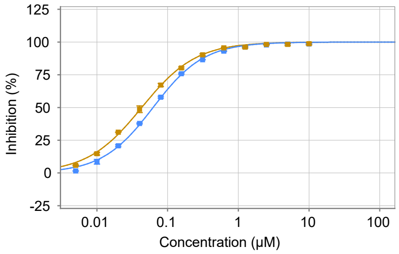

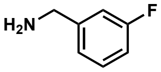

15

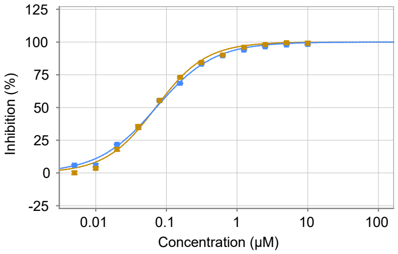

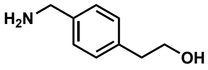

16

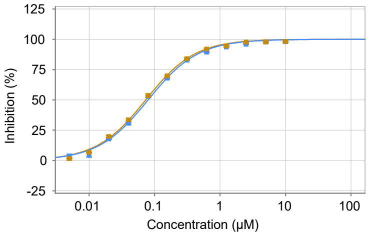

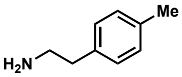

17

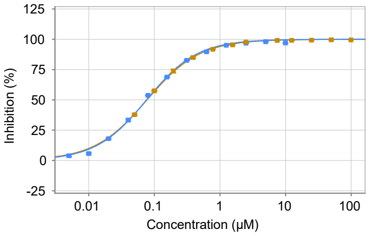

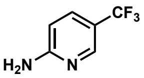

S1

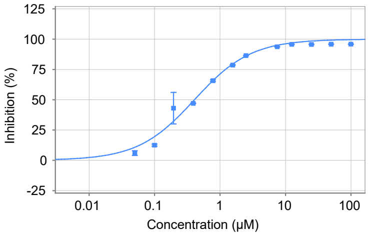

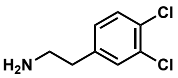

S2

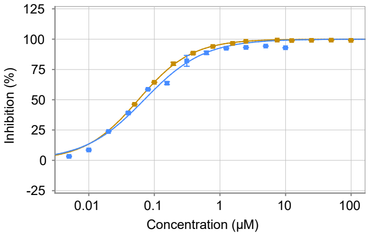

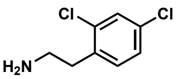

S3

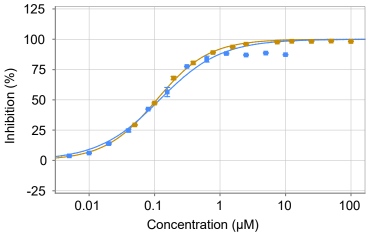

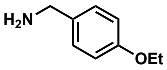

S4

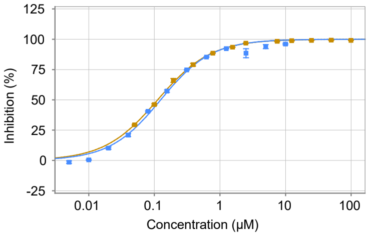

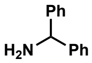

S5

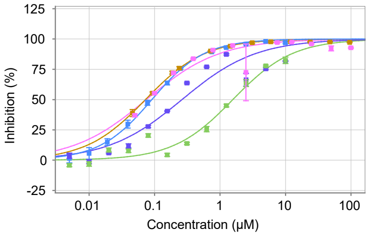

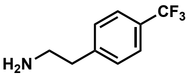

S6

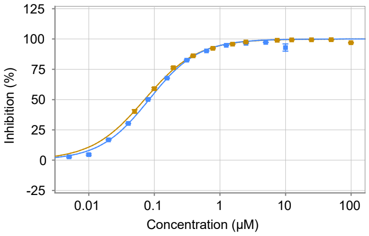

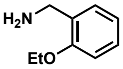

S7

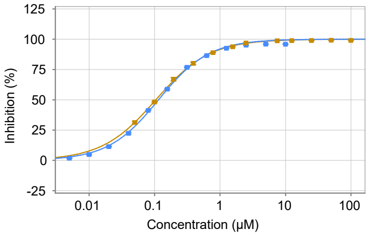

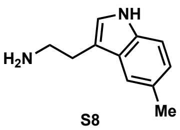

S8

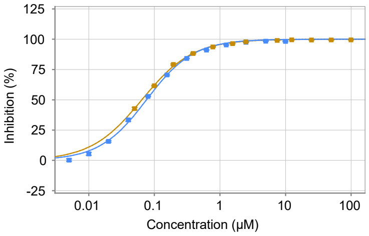

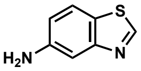

S9

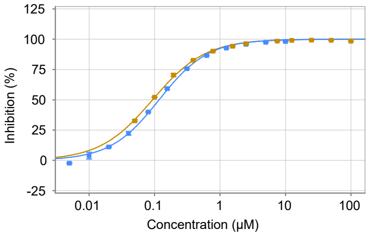

H_2N—Ph

S10

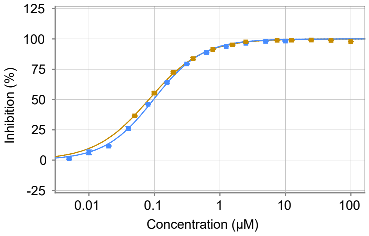

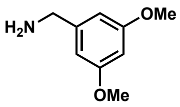

S11

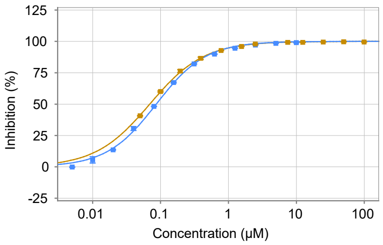

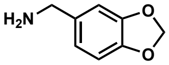

S12

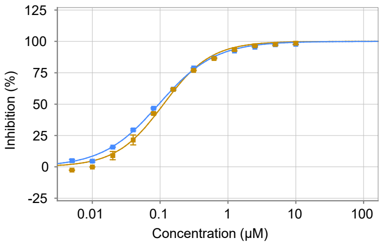

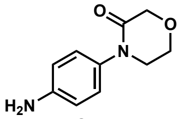

S13

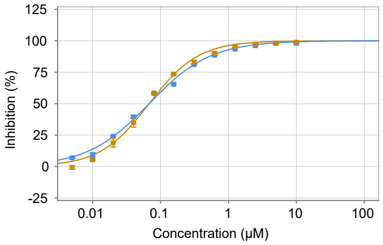

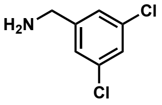

S14

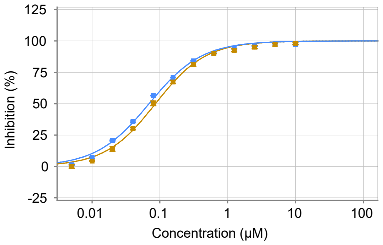

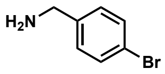

S15

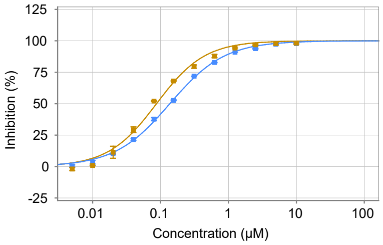

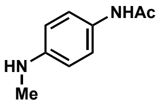

S16

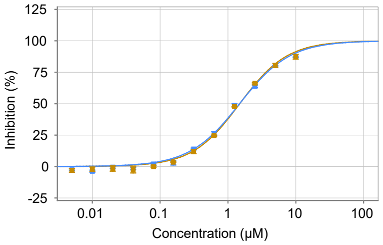

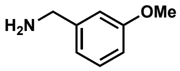

S17

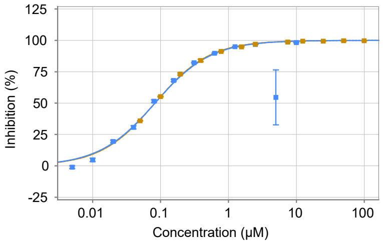

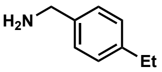

S18

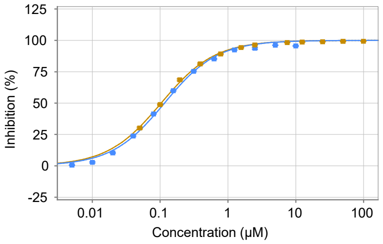

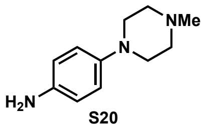

S20

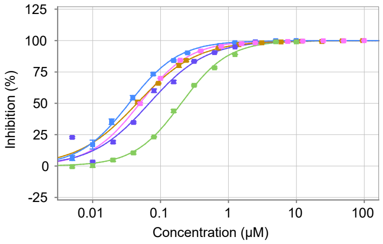

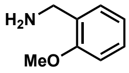

S21

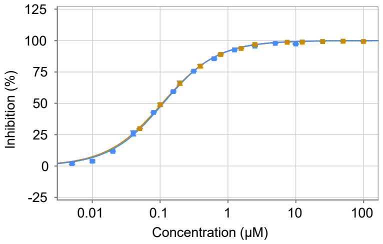

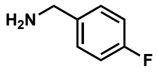

S22

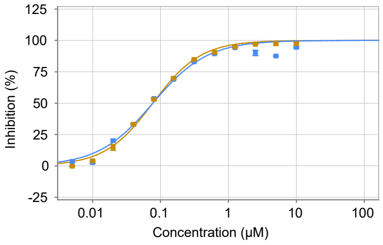

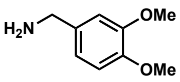

S23

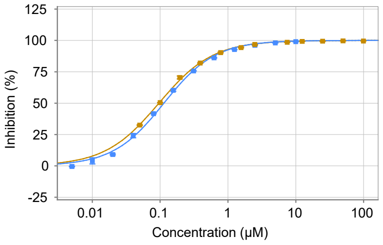

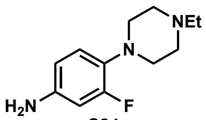

S24

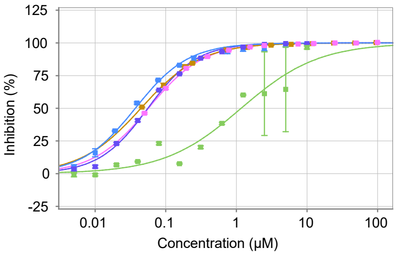

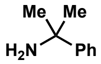

S25

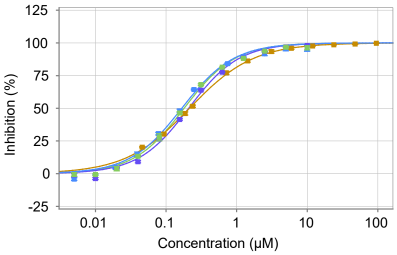

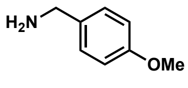

S26

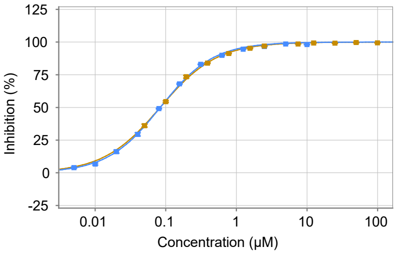

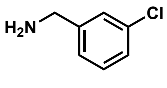

S27

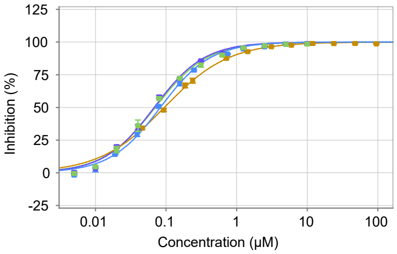

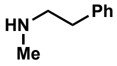

S28

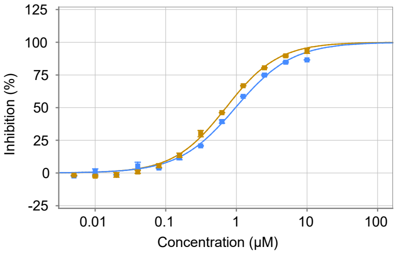

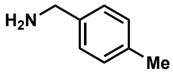

S29

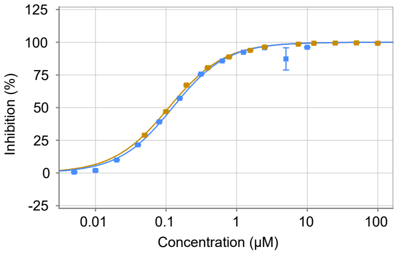

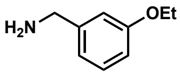

S30

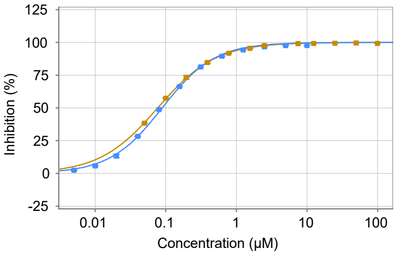

Supplemental References:

1. F. Pedregosa, G. Varoquaux, A. Gramfort, V. Michel, B. Thirion, O. Grisel, M. Blondel, P. Prettenhofer, R. Weiss and V. Dubourg, Scikit-learn: Machine learning in Python, *the Journal of machine Learning research*, 2011, **12**, 2825-2830.
2. M. L. Boby, D. Fearon, M. Ferla, M. Filep, L. Koekemoer, M. C. Robinson, C. M. Consortium‡, J. D. Chodera, A. A. Lee and N. London, Open science discovery of potent noncovalent SARS-CoV-2 main protease inhibitors, *Science*, 2023, **382**, eabo7201.
